# First Case of Mixed Poorly Differentiated Mucinous Adenocarcinoma and High-Grade Neuroendocrine Neoplasm With Amphicrine Differentiation

**DOI:** 10.7759/cureus.114442

**Published:** 2026-08-12

**Authors:** Fnu Raja, Oyedele A Adeyi, Huma Fatima, Berkha Rani, Isam-Eldin A Eltoum

**Affiliations:** 1 Pathology, University of Alabama at Birmingham, Birmingham, USA; 2 Pathology and Laboratory Medicine, University of Alabama at Birmingham, Birmingham, USA; 3 Medicine, Mercy Medical Center, Darby, USA

**Keywords:** amphicrine carcinoma, dual differentiation, electronic microscopic features, gastrointestinal tract, mucinous adenocarcinoma

## Abstract

Amphicrine carcinoma is a rare gastrointestinal carcinoma characterized by dual exocrine and neuroendocrine differentiation within individual tumor cells. Its rarity, overlapping morphological features with those of conventional adenocarcinoma, and evolving classification present diagnostic and therapeutic challenges. We report the rare case of a middle-aged man who presented with dysphagia and weight loss. Imaging revealed a distal esophageal mass, and biopsy confirmed adenocarcinoma. Following neoadjuvant chemoradiation, persistent disease led to esophagogastrectomy. Microscopic examination demonstrated extracellular mucin pools and glandular/cribriform structures, with co-expression of neuroendocrine markers (INSM1, synaptophysin, and chromogranin) and mucin within the same tumor cells. Electron microscopy confirmed the presence of dense-core neurosecretory granules, mucin droplets, and microvilli in the same tumor cells, supporting the diagnosis of amphicrine carcinoma. Amphicrine carcinoma is distinct from conventional adenocarcinoma and mixed neuroendocrine-non-neuroendocrine neoplasms (MiNENs), with prognosis determined by histologic grade. Molecular profiling has revealed recurrent copy-number gains on chromosome 20q13.12-q13.2, involving MYT1, NTSR1, and ZBTB46, suggesting a distinct biological profile. Recognition of amphicrine carcinoma is critical for accurate diagnosis, prognostic assessment, and appropriate treatment. Diagnosis relies on careful evaluation of morphologic features and immunohistochemical findings, while histologic grade appears to predict prognosis. Awareness of this rare entity is essential for appropriate clinical management, and further studies are needed to better define its molecular characteristics and guide the development of targeted therapies.

## Introduction

More than three decades ago, a classification system for neoplasms exhibiting both exocrine and neuroendocrine differentiation was proposed, categorizing them into three groups: mixed (composite) tumors, collision tumors, and amphicrine tumors [1]. The term amphicrine, derived from the Greek meaning “dual secretion,” refers to neoplasms in which individual tumor cells simultaneously exhibit both exocrine (glandular or mucinous) and neuroendocrine differentiation. This contrasts with composite and collision tumors, in which two distinct cellular populations coexist, either intermixed or in proximity [1].

Earlier classifications, including the 2010 WHO framework, grouped amphicrine carcinomas under mixed adenoneuroendocrine carcinomas (MANECs), which were considered intermediate-grade malignancies. Advances in the understanding of histomorphologic features, immunohistochemistry, and electron microscopy have refined the recognition of true dual-phenotype neoplasms, demonstrating mucin droplets, microvilli, and dense-core neuroendocrine granules within the cytoplasm of individual tumor cells [2]. Immunohistochemical evaluation demonstrates co-expression of neuroendocrine markers (chromogranin A, synaptophysin, and INSM1) and epithelial/mucin markers within the same tumor cells.

Reflecting these advances, the 2022 WHO classification recognizes amphicrine carcinoma as a distinct entity, separate from conventional adenocarcinoma and mixed neuroendocrine-non-neuroendocrine neoplasms (MiNENs) [3]. Histologically, amphicrine carcinomas may demonstrate glandular, trabecular, or cribriform architecture, often accompanied by mucin production, signet-ring cell morphology, and neuroendocrine cytoplasmic granules.

Amphicrine carcinoma is exceedingly rare and has been reported at various gastrointestinal sites. Clinical presentation, endoscopic findings, and imaging features are nonspecific, emphasizing that histopathologic evaluation with immunohistochemical confirmation remains the gold standard for diagnosis.

## Case presentation

A middle-aged man with no prior history of esophageal malignancy presented with several weeks of progressive intolerance to oral intake and unintentional weight loss. Computed tomography (CT) revealed a mass involving the gastroesophageal junction (GEJ). Esophagogastroduodenoscopy (EGD) with biopsy confirmed esophageal adenocarcinoma.

The patient underwent neoadjuvant chemoradiation with a total radiation dose of 58 Gy. Follow-up EGD revealed persistent disease. Esophagogastrectomy with regional lymph node dissection was subsequently performed.

**Figure 1:**
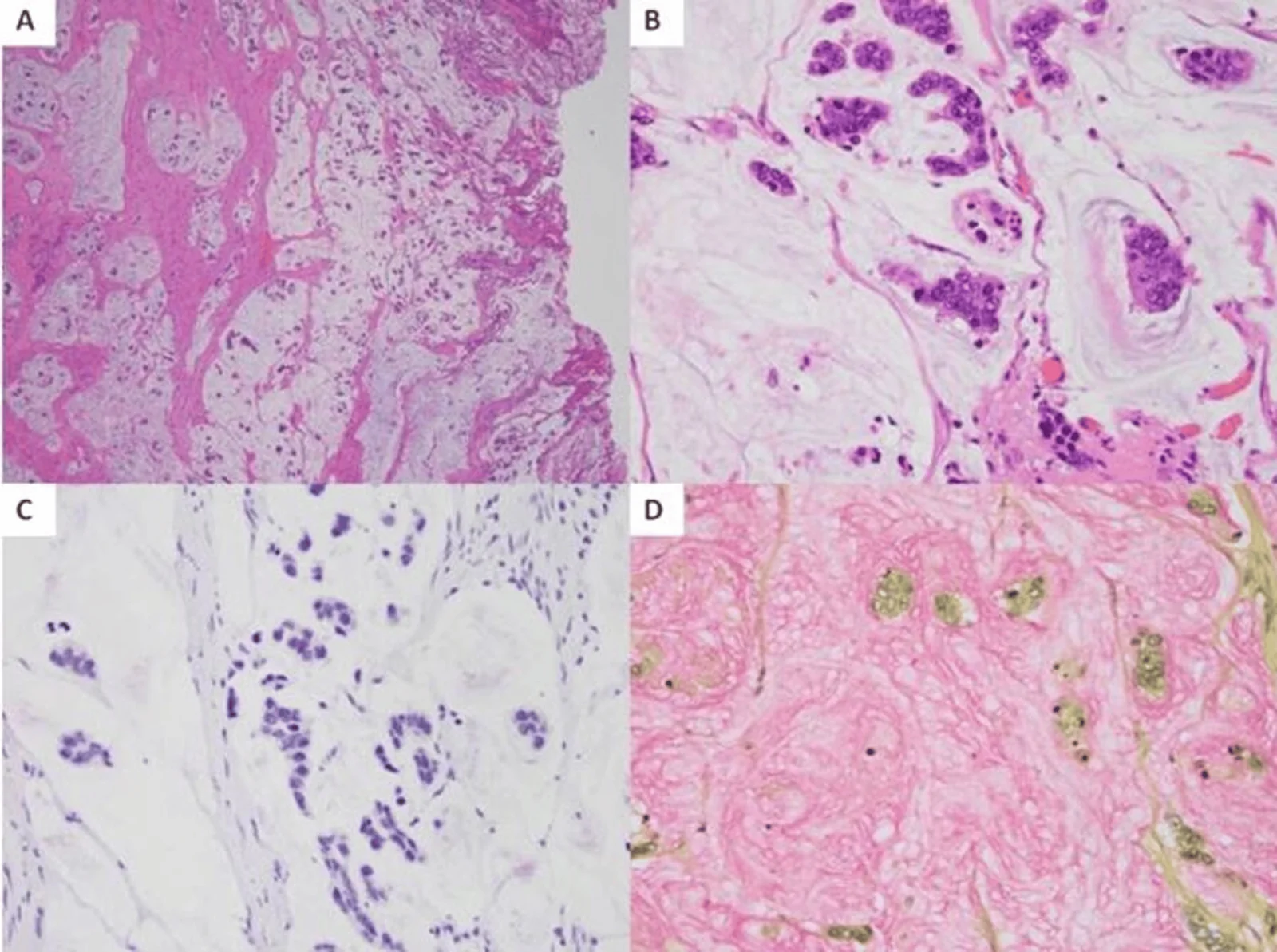
Histopathologic features of the tumor. (A) Low-power hematoxylin and eosin (H&E) section showing abundant extracellular mucin pools. (B) High-power view demonstrating floating malignant epithelial cells within mucin. (C) Immunohistochemical staining for INSM1 showing lack of expression in tumor cells. (D) Mucicarmine stain highlighting extracellular mucin.

**Figure 2:**
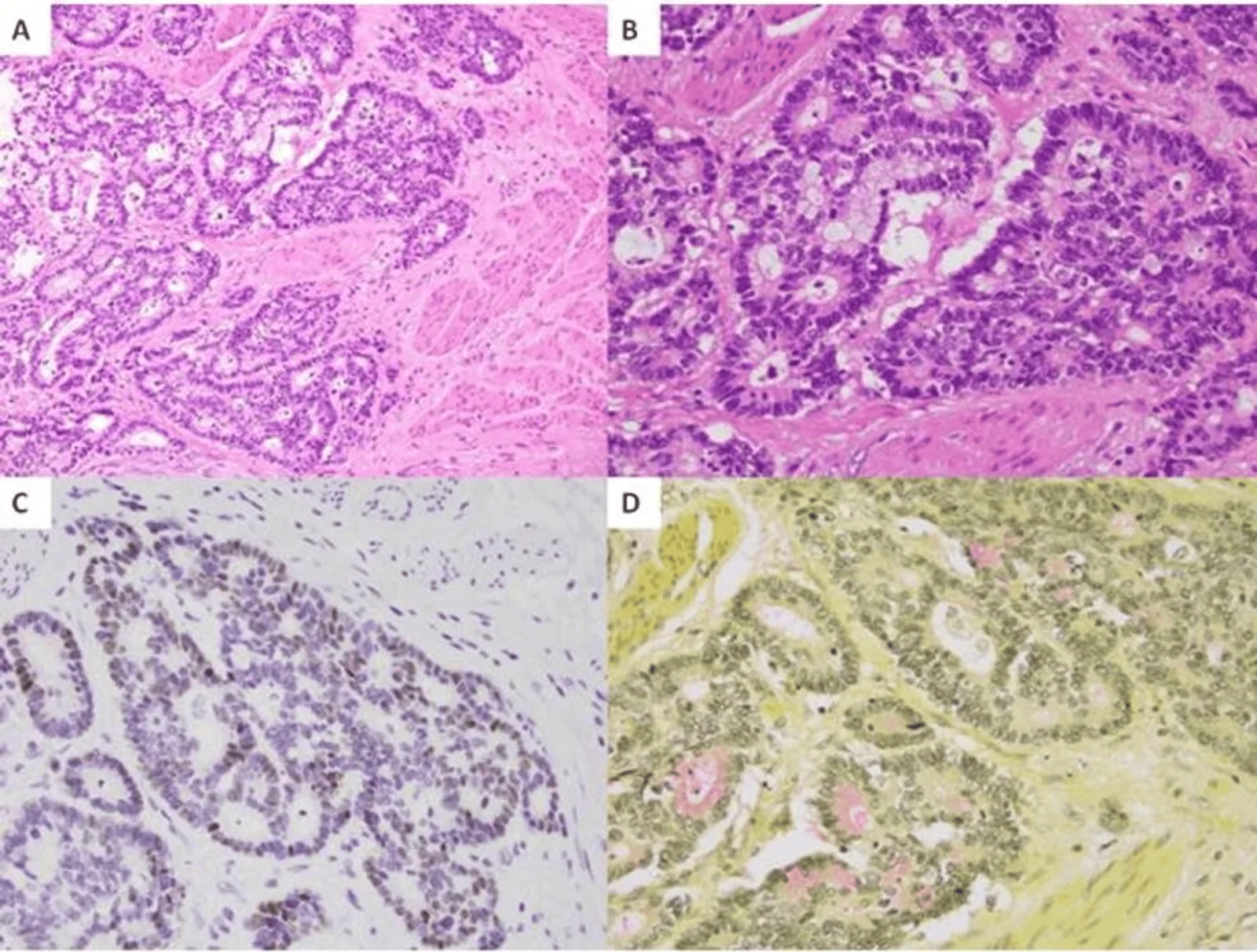
Histopathologic features of the tumor. (A) Low-power hematoxylin and eosin (H&E) section showing infiltrative carcinoma with glandular and cribriform architecture. (B) High-power view demonstrating intraluminal mucin and tumor cells with salt-and-pepper chromatin. (C) Immunohistochemical staining for INSM1 showing positive expression in tumor cells. (D) Mucicarmine stain highlighting mucin.

**Figure 3:**
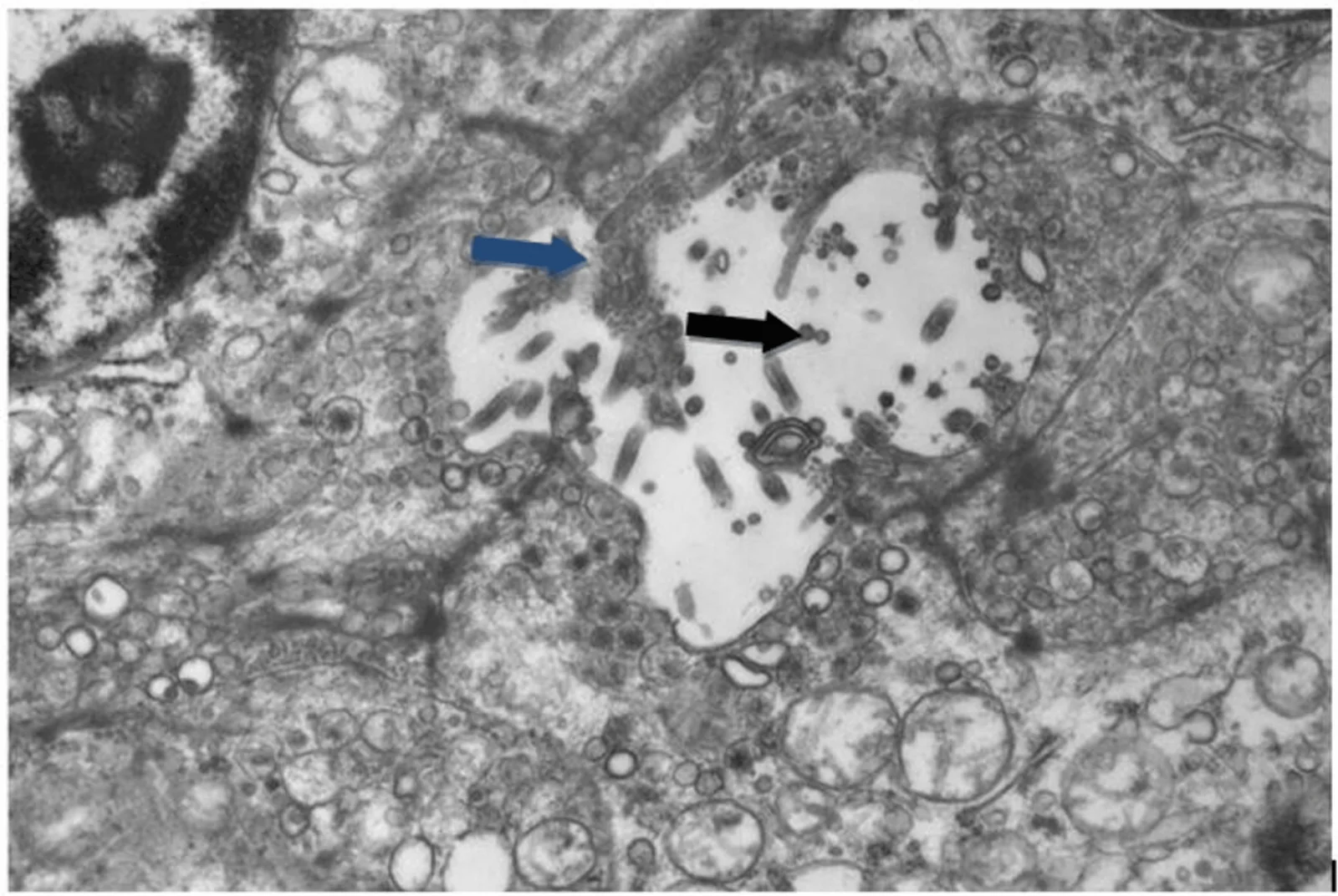
Electron microscopy of tumor cells demonstrating dual differentiation. A single tumor cell shows microvilli projecting into the lumen (glandular differentiation, blue arrow) and apical dense-core granules (neuroendocrine differentiation, black arrow). The cell contains both membrane-bound neuroendocrine granules and mucin droplets, consistent with combined neuroendocrine and mucinous differentiation.

Gross examination identified a 3.4-cm ulcerated, infiltrative mass located 0.9 cm proximal to the GEJ. Microscopic evaluation revealed two distinct morphologic patterns: infiltrative pools of extracellular mucin containing malignant epithelial cells and adjacent glandular/cribriform structures with intraluminal mucin and “salt-and-pepper” chromatin. Immunohistochemical evaluation demonstrated co-expression of neuroendocrine markers (INSM1, synaptophysin, and chromogranin) and mucin within the same tumor cells (Figures 1, 2). The Ki-67 proliferation index was >30%. Electron microscopy confirmed dual differentiation, demonstrating dense-core neurosecretory granules and electron-lucent mucin droplets within individual tumor cells (Figure 3). Based on the combined morphologic, immunohistochemical, and ultrastructural findings, the final diagnosis of mixed poorly differentiated adenocarcinoma (mucinous) and high-grade neuroendocrine neoplasm with amphicrine features was rendered.

## Discussion

Amphicrine carcinoma is a rare gastrointestinal malignancy characterized by simultaneous exocrine and neuroendocrine differentiation within individual tumor cells [1]. The concept was first introduced by Feyrter in 1938 [2] and subsequently refined by Lewin and Appelman in 1987, who distinguished amphicrine tumors from mixed and collision neoplasms [3]. Despite these early classification frameworks, the classification of amphicrine carcinoma has remained inconsistent, particularly for non-appendiceal lesions of the stomach and intestine, contributing to diagnostic challenges [4,5]. Misdiagnosis as conventional adenocarcinoma, particularly signet-ring cell carcinoma, may occur when neuroendocrine markers are not included in the immunohistochemical evaluation [5].

Pure amphicrine carcinomas are rare, and mixed forms may be underrecognized, underscoring the importance of careful histopathologic evaluation and appropriate immunohistochemical assessment [1].

Prognosis is closely associated with histologic grade. Low-grade tumors typically exhibit tubular architecture, low cytologic atypia, and low mitotic activity, whereas high-grade tumors demonstrate infiltrative growth, increased proliferative activity, and more aggressive behavior [1,6]. Although the prognostic value of the Ki-67 proliferation index remains debated, studies suggest that increased mitotic activity and architectural complexity are associated with poorer outcomes [4,6].

Molecular profiling further supports the biological distinctiveness of amphicrine carcinoma. Gastric amphicrine carcinomas have demonstrated recurrent copy number gains at chromosome 20q13.12-q13.2 involving MYT1, NTSR1, and ZBTB46, genes implicated in neuroendocrine differentiation and oncogenic signaling [7]. MYT1 regulates endocrine lineage commitment, NTSR1 promotes neuroendocrine differentiation, and ZBTB46 activates STAT3 signaling, contributing to neuroendocrine-like features [7]. These findings support the concept that amphicrine carcinoma represents a distinct neoplastic entity rather than a simple admixture of adenocarcinoma and neuroendocrine carcinoma.

Amphicrine carcinoma is molecularly distinct from MiNENs. Although some oncogenic pathways, including TP53 mutations, may overlap, copy number variation patterns differ significantly, supporting the WHO's 2022 classification of amphicrine carcinoma as distinct from MiNENs [8]. Recognition of this entity has important prognostic and potential therapeutic implications. Emerging evidence also suggests a possible role for sex hormone-related signaling; however, further studies are needed to clarify its biological and therapeutic significance [8].

## Conclusions

Amphicrine carcinoma of the gastrointestinal tract is a rare and distinct entity characterized by dual exocrine and neuroendocrine differentiation within individual tumor cells. Its overlapping morphologic features with conventional adenocarcinoma and MiNENs can pose diagnostic challenges, highlighting the importance of immunohistochemical and ultrastructural evaluation. Histologic grade remains an important prognostic determinant, while molecular studies suggest biological similarities to conventional adenocarcinoma, with recurrent genomic alterations involving MYT1, NTSR1, and ZBTB46. Accurate recognition of amphicrine carcinoma is essential for appropriate prognostic assessment and therapeutic decision-making. Further molecular and clinical studies are warranted to elucidate its biology, identify potential targeted therapeutic strategies, and improve outcomes for patients with this rare malignancy.
